# Association of an Automated Blood Pressure Measurement Quality Improvement Program With Terminal Digit Preference and Recorded Mean Blood Pressure in 11 Clinics

**DOI:** 10.1001/jamanetworkopen.2022.29098

**Published:** 2022-08-31

**Authors:** Thomas E. Kottke, Jeffrey P. Anderson, Jacob D. Zillhardt, JoAnn M. Sperl-Hillen, Patrick J. O’Connor, Beverly B. Green, Rae Ann Williams, Beth M. Averbeck, Michael N. Stiffman, MarySue Beran, Michael Rakotz, Karen L. Margolis

**Affiliations:** 1 HealthPartners Institute, Minneapolis, Minnesota; 2 Kaiser Permanente Washington Health Research Institute, Seattle; 3 HealthPartners, Minneapolis, Minnesota; 4 Park Nicollet Health Services, Saint Louis Park, Minnesota; 5 American Medical Association, Chicago, Illinois

## Abstract

**Importance:**

Terminal digit preference has been shown to be associated with inaccurate blood pressure (BP) recording.

**Objective:**

To evaluate whether converting from manual BP measurement with aneroid sphygmomanometers to automated BP measurement was associated with terminal digit preference, mean levels of recorded BP, and the rate at which hypertension was diagnosed.

**Design, Setting, and Participants:**

This quality improvement study was conducted from May 9, 2021, to March 24, 2022, using interrupted time series analysis of medical record data from 11 primary care clinics in a single health care system from April 2008 to April 2015. The study population was patients aged 18 to 75 years who had their BP measured and recorded at least once during the study period.

**Exposures:**

Manual BP measurement before April 2012 vs automated BP measurement with the Omron HEM-907XL monitor from May 2012 to April 2015.

**Main Outcomes and Measures:**

The main outcome was the distribution of terminal digits and mean systolic BP (SBP) values obtained during 4 years of manual measurement vs 3 years of automated measurement, assessed using a generalized linear mixed regression model with a random intercept for clinic and adjusted for seasonal fluctuations and patient demographic and clinical characteristics.

**Results:**

The study included 1 541 227 BP measurements from 225 504 unique patients during the entire study period, with 849 978 BP measurements from 165 137 patients (mean [SD] age, 47.1 [15.2] years; 58.2% female) during the manual measurement period and 691 249 measurements from 149 080 patients (mean [SD] age, 48.4 [15.3] years; 56.3% female) during the automated measurement period. With manual measurement, 32.8% of SBP terminal digits were 0 (20% was the expected value because nursing staff was instructed to record BP to the nearest even digit). This proportion decreased to 12.4% during the automated measurement period (expected value, 10%) when both even and odd digits were to be recorded. After automated measurement was implemented, the mean SBP estimated with statistical modeling increased by 5.09 mm Hg (95% CI, 4.98-5.19 mm Hg). Fewer BP values recorded during the automated than the manual measurement period were below 140/90 mm Hg (69.9% vs 84.3%; difference, –14.5%; 95% CI, –14.6% to –14.3%) and below 130/80 mm Hg (42.1% vs 60.0%; difference, –17.9%; 95% CI, –18.0% to –17.7%). The proportion of patients with a diagnosis of hypertension was 4.3 percentage points higher (23.4% vs 19.1%) during the automated measurement period.

**Conclusions and Relevance:**

In this quality improvement study, automated BP measurement was associated with decreased terminal digit preference and significantly higher mean BP levels. The method of BP measurement was also associated with the rate at which hypertension was diagnosed. These findings may have implications for pay-for-performance programs, which may create an incentive to record BP levels that meet a particular goal and a disincentive to adopt automated measurement of BP.

## Key Points

**Question:**

Is converting from manually measured blood pressure (BP) to automated monitoring associated with terminal digit preference, mean recorded BP levels, and hypertension diagnosis rate?

**Findings:**

In this quality improvement study of 1 541 227 BP measurements from 225 504 patients, when aneroid sphygmomanometers were replaced with automated monitors, terminal digit preference decreased, mean systolic BP immediately increased, and during the subsequent 3 years, the proportion of patients with the diagnosis of hypertension increased from 19.1% to 23.4%.

**Meaning:**

The findings suggest that the method of measuring BP is associated with mean BP levels and the extent to which hypertension is diagnosed.

## Teaser

This quality improvement study examines differences in terminal digit preferences, mean recorded blood pressure levels, and diagnoses of hypertension when blood pressure is measured manually vs with an automated monitor.

## Introduction

The difficulty of accurately assessing blood pressure (BP) has been recognized by both epidemiologists and clinicians for more than 60 years,^1,2,3,4^ and terminal digit preference is a recognized indicator of erroneous BP recording.^5,6,7,8,9,10,11^ Multiple studies have reported that terminal digit preference is associated with biased observations^4,6,7,8,12^ or with fewer patients being identified as having hypertension.^6,9^

To improve accuracy and quality of BP recording, HealthPartners launched a BP assessment reliability program in its primary care clinics in 2006. The organization implemented a training program for nursing personnel that emphasized the importance of correct measurement technique, including proper patient positioning, using a cuff of the appropriate size, allowing the patient to rest before measurement, and assuring that the equipment was calibrated and in good condition. It also equipped all primary care examination rooms with wall-mounted Welch Allyn aneroid sphygmomanometers. We refer to this time as the manual measurement period.

Because of persisting terminal digit preference and beliefs that use of automated monitors would result in lower BP values on average, HealthPartners began replacing the sphygmomanometers with automated devices (Omron HEM-907XL, Omron Healthcare) in all its primary care clinics in 2012. HealthPartners also further strengthened its training program for nursing personnel. We refer to this time as the automated measurement period.

Mean BP values were higher in 27 of 28 HealthPartners clinics after the deployment of automated monitors (eTable 1 in the Supplement), and hypertension control rates, as reported to Minnesota Community Measurement, declined from over 80% in 2011 to 73.5% in 2013.^13,14^ In this study, we assessed the distribution of terminal digits, the distribution of BP values, and the difference in mean BP values during the final 4 years of the manual measurement period and the first 3 years of the automated measurement period at 11 HealthPartners primary care clinics that converted to automated measurement in April 2012.

## Methods

For this quality improvement study, we used the Standards for Quality Improvement Reporting Excellence (SQUIRE) reporting guideline.^15^ At each patient’s first clinic visit in each calendar year, HealthPartners invited the patient to give or decline written informed consent to use their medical records for purposes of research. The records of patients who ever declined were excluded from this analysis. The HealthPartners institutional review board determined that this study was quality improvement and thus not subject to institutional review board oversight.

### Patient Population

The study population included patients aged 18 to 75 years who had their BP measured and recorded at least once in 1 of 11 primary care clinics between April 2008 and April 2015. Data were analyzed from May 9, 2021, to March 24, 2022.

Systolic BP (SBP) values less than 50 mm Hg or greater than 250 mm Hg and diastolic BP (DBP) values less than 20 mm Hg or greater than 150 mm Hg were considered invalid and removed from the analytic data set. Our analysis was based on the remaining values obtained from patients during the manual measurement period (April 2008 to March 2012) and the automated measurement period (May 2012 to April 2015). Primary analyses used all available measures except when specified as only the last measure per patient encounter. We considered a patient to have hypertension if 1 or more of the following codes were associated with or recorded before an encounter: *International Classification of Diseases, Ninth Revision, Clinical Modification* codes 401.0, 401.1, and 401.9 or *International Statistical Classification of Diseases, Tenth Revision, Clinical Modification* code I10.

### Selection of Clinics for the Analysis

Blood pressure levels increase and decrease in seasonal cycles,^16,17,18,19^ and the intensity of treatment may need to vary by season.^20^ Therefore, we chose to avoid the statistical modeling challenges associated with seasonal BP cycles by limiting the analysis to the 11 clinics that began to use automated BP measurement in April 2012.

### Instructions to Nursing Staff Measuring BP

Beginning in May 2006, the nursing staff was instructed to use a recently calibrated aneroid manometer with the aneroid dial at eye level, select the appropriate cuff size based on the circumference of the middle portion of the upper arm, and use the bell of the stethoscope for auscultation. The patient was to sit quietly for a period at rest before measurement, with both feet flat on the floor and the back supported. The BP cuff was to be placed around the bare arm, which was supported or allowed to rest on a solid surface so the inner aspect of the bend of the elbow would be level with the heart. The nursing staff was given detailed instructions regarding placement of the cuff and performance of an initial palpatory estimate of SBP followed by an auscultatory assessment during which the cuff was to be deflated at 2 to 3 mm Hg per second. Exact readings to the nearest even digit were to be recorded. If the BP was 130/80 mm Hg or above, it was to be remeasured after 5 minutes of quiet rest. If the patient had initially waited quietly for 5 minutes, BP was to be remeasured after 1 to 2 minutes.

Other than instruction on how to operate the automated monitor, the instructions to the nursing staff during the automated measurement period were similar to the instructions given during the manual measurement period. If the first reading was 130/80 mm Hg or above, the nursing staff was to have the patient rest quietly for 5 minutes and then repeat the measurement. The nursing staff was permitted to use the manual mode on the automated monitor and a stethoscope to obtain the BP readings if error messages were obtained with the fully automated mode.

### Statistical Analysis

We first generated descriptive summaries of patient characteristics and BP values for the manual and automated measurement periods. Owing to the large study sample, we did not report *P* values because statistical significance was consistently achieved even for differences that were too small to be considered clinically meaningful. We then grouped BP values by month and conducted an interrupted time series analysis, with the inflection point at April 2012, when all 11 study clinics implemented automatic measurement.^21,22,23,24^ To adjust for seasonal fluctuation in BP levels, we fit a generalized linear mixed regression model with a random intercept for clinic and adjusted for patient age (continuous), body mass index (calculated as weight in kilograms divided by height in meters squared; <25, 25-29, ≥30, or missing), sex (female or male), self-reported ethnicity (Hispanic, non-Hispanic, or missing), insurance status (Medicaid or government assistance, Medicare, commercial, or other or unknown), self-reported race (Asian, Black, White, or other or unknown [American Indian or Alaska Native, Hispanic or Latino, Native Hawaiian or other Pacific Islander, or some other race]), hypertension (yes or no), Charlson Comorbidity Index score (continuous), and Fourier terms^21,22^ for sinusoidal oscillation by season. Primary results were reported as β estimates representing comparative changes in mm Hg, with 95% CIs. All analyses were conducted using SAS, version 9.4 (SAS Institute Inc) or R, version 4.0.3 (R Project for Statistical Computing).

## Results

### Demographic Characteristics of Patients

The study included 1 541 227 BP measurements from 225 504 unique patients during the entire study period, with 849 978 BP measurements from the records of 165 137 patients (mean [SD] age, 47.1 [15.2] years; 58.2% female) during the manual measurement period and 691 249 measurements from 149 080 patients (mean [SD] age, 48.4 [15.3] years; 56.3% female) during the automated measurement period. During the manual measurement period, 19.1% of the patients received a diagnosis of hypertension, and 23.4% received a diagnosis of hypertension during the automated measurement period (difference, 4.3 percentage points). In all age groups, the proportion of patients with a diagnosis of hypertension was higher during the automated measurement period (eTable 2 in the Supplement). There were more BP measurements per patient during the longer, manual measurement period (mean [SD], 3.6 [5.2] vs 2.6 [3.8]) and fewer BP measurements per encounter during the manual measurement period (mean [SD], 1.0 [0.3] vs 1.2 [0.5]) (Table 1).

**Table 1.** Distribution of BP Measurements by Patient Demographic Characteristics During the 2 Measurement Periods in 11 Primary Care Clinics

Characteristic	BP measurements^a^	
	Manual measurement period	Automated measurement period
Patients, No.	165 137	149 080
BP measurements, No.	849 978	691 249
BP measurements per patient, mean (SD)	3.6 (5.2)	2.6 (3.8)
BP measurements per encounter, mean (SD)	1.0 (0.3)	1.2 (0.5)
Age, mean (SD), y	47.1 (15.2)	48.4 (15.3)
Sex		
Female	494 687 (58.2)	389 173 (56.3)
Male	355 291 (41.8)	302 076 (43.7)
Race		
Asian	54 399 (6.4)	51 844 (7.5)
Black or African American	124 947 (14.7)	109 909 (15.9)
White	617 934 (72.7)	484 566 (70.1)
Other race^b^	40 799 (4.8)	35 945 (5.2)
Unknown	11 900 (1.4)	8986 (1.3)
Ethnicity		
Hispanic	17 850 (2.1)	18 664 (2.7)
Non-Hispanic	498 937 (58.7)	458 298 (66.3)
Unknown	333 191 (39.2)	214 978 (31.1)
Insurance type		
Commercial	526 986 (62.0)	391 938 (56.7)
Medicare	127 497 (15.0)	125 116 (18.1)
Medicaid or government assistance	163 196 (19.2)	155 531 (22.5)
Other or no insurance	32 299 (3.8)	18 664 (2.7)
Body mass index		
Mean (SD)^c^	29.9 (7.1)	30.1 (7.2)
<18.5	8500 (1.0)	5530 (0.8)
18.5 to <25.0	175 945 (20.7)	135 485 (19.6)
25.0 to <30.0	223 544 (26.3)	175 577 (25.4)
30.0 to <35.0	158 096 (18.6)	120 969 (17.5)
35.0 to <40.0	85 848 (10.1)	62 212 (9.0)
≥40.0	66 298 (7.8)	49 079 (7.1)
Missing	131 747 (15.5)	142 397 (20.6)
Charlson Comorbidity Index score		
Mean (SD)	1.3 (1.6)	1.7 (1.9)
0	358 691 (42.2)	244 702 (35.4)
1-2	330 641 (38.9)	264 057 (38.2)
3-4	118 147 (13.9)	122 351 (17.7)
≥5	42 499 (5.0)	60 139 (8.7)

Abbreviation: BP, blood pressure.

^a^ Data are presented as the number (percentage) of BP measurements unless otherwise indicated. The manual measurement period was from April 2008 to March 2012, and the automated measurement period was from May 2012 to April 2015.

^b^ Other race included American Indian or Alaska Native, Hispanic or Latino, Native Hawaiian or other Pacific Islander, or some other race.

^c^ Calculated as weight in kilograms divided by height in meters squared.

Table 1 shows the patient characteristics associated with all BP measurements in each period. The mean patient age and the proportion of females were higher during both periods because female patients and those who were older were more likely to have multiple measurements. Of 1 541 227 total BP measurements, 6.9% were in patients who self-identified as Asian, 15.2% in Black or African American patients, 71.5% in White patients, 5.0% in patients who reported other race, and 1.4% in patients with unknown race. Although the ethnicity of the patients was unknown for 35.6% of measurements, only 2.4% were in patients who self-identified as Hispanic; 62.1% were in non-Hispanic patients. By insurance type, 59.6% of measurements were in patients insured with a commercial insurance product, 16.4% in patients with Medicare, and 20.7% in patients with Medicaid or another governmental assistance program. The mean (SD) body mass index was 29.9 (7.1). The mean (SD) Charlson Comorbidity Index score was 1.3 (1.6) during the manual measurement period and 1.7 (1.9) during the automated measurement period.

### Terminal Digit Preference Before and After Adoption of Automated Measurement

**Figure 1.**
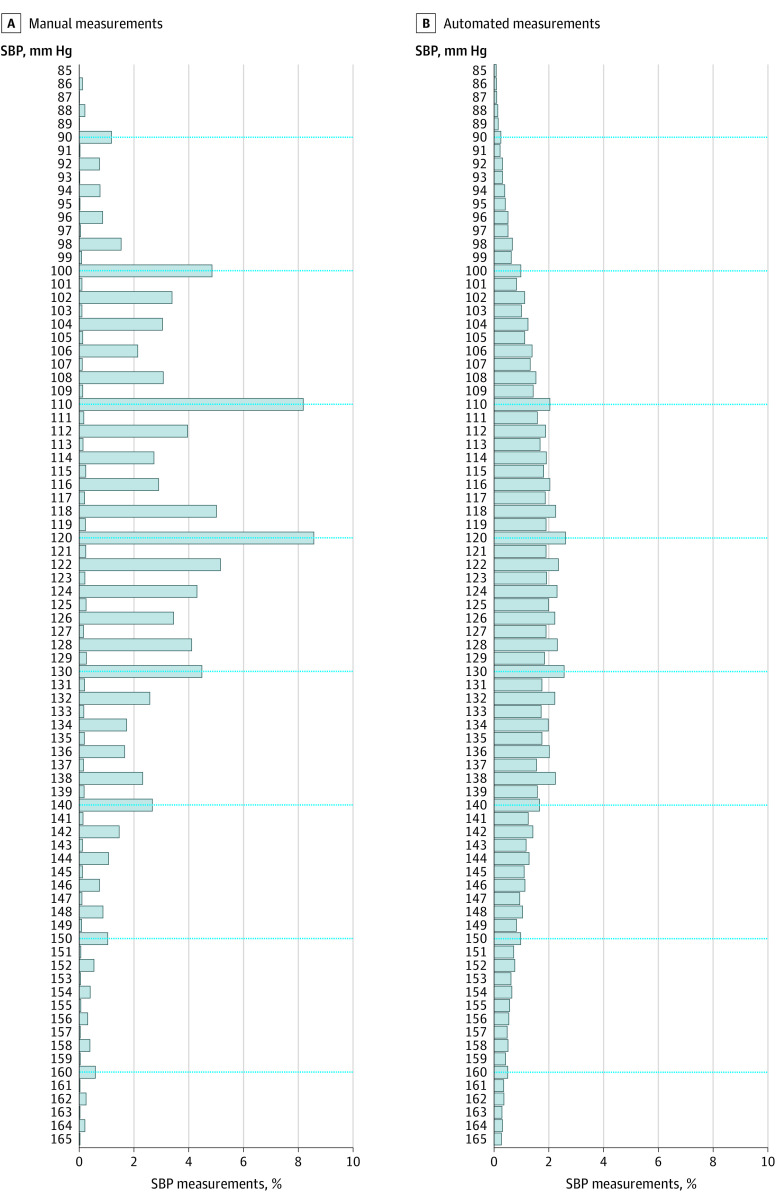
Distribution of Systolic Blood Pressure (SBP) Values Recorded During the Manual Measurement Period and the Automated Measurement Period at 11 HealthPartners Clinics From 2008 to 2015 There were 849 978 SBP measurements during the manual measurement period (April 2008 to March 2012) and 691 249 during the automated measurement period (May 2012 to April 2015).

Terminal digit preference and the preference to record certain SBP values were greater during the manual measurement period than during the automated measurement period (Figure 1). During the period of manual measurement, when the nursing assistants were trained to read the BP to an even terminal digit and the prevalence of each terminal digit would be expected to be 20%, 32.8% of all terminal digits were 0. During the automated measurement period, 12.4% were 0 instead of the expected 10%. The prevalence of 0 as the terminal digit decreased from 14.6% of readings in the first year of the automated measurement period to 10.8% in the last year analyzed (eFigure 1 in the Supplement).

A preference for certain SBP values was also found. In addition to values ending in 0, an SBP of 138 mm Hg was more prevalent than expected among all patients during the automated measurement period (Figure 1) and particularly for the last measurement of an encounter among patients with hypertension during the period of automated measurement (eFigure 2 in the Supplement).

### Mean SBP Values During the Manual and Automated Measurement Periods

**Figure 2.**
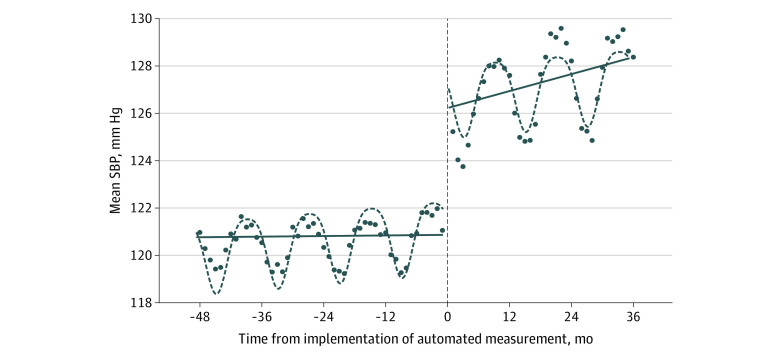
Mean Systolic Blood Pressure (SBP) Levels by Month Before and After Implementation of Automated Monitoring The manual measurement period was from months −48 to 0, and the automated measurement period from months 0 to 36. Month 0 was April 2012. The upward trend in mean SBP values during the automated measurement period is consistent with delayed use of automated measurement. Circles indicate mean values; dashed vertical line, implementation of automated measurement; dashed curved lines, season-adjusted fitted regression; and solid lines, period-specific linear trend.

After adjustment for covariates, the increase in the mean SBP value at the onset of the automated measurement period was 5.09 mm Hg (95% CI, 4.98-5.19 mm Hg) (Figure 2 and Table 2). The mean annual change in SBP was −0.06 mm Hg (95% CI, –0.09 to –0.03 mm Hg) during the manual measurement period and 0.47 mm Hg (95% CI, 0.42-0.51 mm Hg) during the automated measurement period (Table 2).

**Table 2.** Adjusted Annual Change in SBP and Estimated Change at Implementation of Automated Measurement^a^

	Value (95% CI), mm Hg
Annual change in SBP	
Manual measurement period	–0.06 (–0.09 to –0.03)
Automated measurement period	0.47 (0.42 to 0.51)
Estimated change at implementation of automated measurement	5.09 (4.98 to 5.19)

Abbreviation: SBP, systolic blood pressure.

^a^ Generalized linear mixed model with a random intercept for clinic and adjusted for patient age (continuous), body mass index (calculated as weight in kilograms divided by height in meters squared; <25, 25-29, ≥30, or missing), sex (female, male), ethnicity (Hispanic, non-Hispanic, or missing), insurance status (Medicaid or government assistance, Medicare, commercial, or other or unknown), race (Asian, Black, White, or other or unknown), hypertension (yes or no), Charlson Comorbidity Index score (continuous), and Fourier terms for sinusoidal oscillation by season.

Without adjustment for covariates, the mean DBP was 5.3 mm Hg (95% CI, 5.2-5.3 mm Hg) higher during the automated measurement period than during the manual measurement period. The mean SBP value was 6.6 mm Hg (95% CI, 6.5-6.6 mm Hg) higher (Table 3). Stratified by season, both mean SBP and mean DBP were lowest in summer and highest in winter, with intermediate values in spring and fall (Table 3 and Figure 2). The difference in mean values between the manual measurement period and the automated measurement period ranged from 5.3 mm Hg (95% CI, 5.2-5.4 mm Hg) in summer to 7.4 mm Hg (95% CI, 7.3-7.6 mm Hg) in winter.

**Table 3.** Mean Unadjusted BP Values by Measurement Period and Season at 11 Primary Care Clinics From 2008 to 2015

	Manual measurement period	Automated measurement period	Difference (95% CI)
BP measurements, No.	849 978	691 249	NA
BP, mean (SD)			
Diastolic	73.4 (10.6)	78.6 (12.5)	5.3 (5.2 to 5.3)
Systolic^a^			
Overall	120.6 (16.5)	127.1 (18.8)	6.6 (6.5 to 6.6)
Spring	120.5 (16.6)	127.5 (18.8)	7.0 (6.9 to 7.1)
Summer	119.5 (16.2)	124.7 (18.6)	5.3 (5.2 to 5.4)
Fall	120.8 (16.6)	127.3 (18.8)	6.4 (6.3 to 6.6)
Winter	121.5 (16.6)	128.9 (18.9)	7.4 (7.3 to 7.6)
BP cutoff, No. (%)			
<140/90 mm Hg	716 531 (84.3)	483 183 (69.9)	–14.5 (–14.6 to –14.3)
<130/80 mm Hg	509 987 (60.0)	291 016 (42.1)	–17.9 (–18.0 to –17.7)
Last BP measurements per patient encounter, No.	808 238	577 192	NA
Last BP per patient encounter, mean (SD)			
Diastolic	72.9 (10.2)	76.7 (11.3)	3.7 (3.7 to 3.8)
Systolic^a^			
Overall	119.5 (15.6)	123.5 (16.7)	4.0 (3.9 to 4.0)
Spring	119.5 (15.7)	123.8 (16.5)	4.2 (4.1 to 4.3)
Summer	118.6 (15.4)	121.6 (16.5)	3.0 (2.9 to 3.1)
Fall	119.8 (15.6)	123.7 (16.7)	3.9 (3.8 to 4.0)
Winter	120.3 (15.7)	125.0 (16.8)	4.7 (4.6 to 4.8)
BP cutoff, No. (%)			
<140/90 mm Hg, No. (%)	702 359 (86.9)	470 411 (81.5)	–5.4 (–5.5 to –5.3)
<130/80 mm Hg, No. (%)	504 341 (62.4)	285 133 (49.4)	–12.9 (–13.1 to –12.8)

Abbreviations: BP, blood pressure; NA, not applicable.

^a^ Spring was from March to May; summer, June to August; fall, September to November; and winter, December to February.

When all BP values were included, fewer obtained during the automated measurement period than the manual measurement period were below 140/90 mm Hg (69.9% vs 84.3%; difference, –14.5%; 95% CI, –14.6% to –14.3%) (Table 3). Likewise, fewer BP values obtained during the automated measurement period were below 130/80 mm Hg (42.1% vs 60.0%; difference, –17.9%; 95% CI, –18.0% to –17.7%). When only the last BP value recorded during a patient encounter (95.1% of patients during the manual measurement period and 83.5% during the automated measurement period had only 1 measurement) was considered, fewer BP values obtained during the automated measurement period were below 140/90 mm Hg (81.5% vs 86.9%; difference, –5.4%; 95% CI, –5.5% to –5.3%) and below 130/80 mm Hg (49.4% vs 62.4%; difference, –12.9%; 95% CI, –13.1% to –12.8%).

## Discussion

In this quality improvement study, we analyzed 1 541 227 BP values recorded 4 years before and 3 years after 11 primary care clinics began using automated monitors in a quality improvement initiative to improve the reliability of BP assessment. The mean SBP increased by 5.09 mm Hg immediately after implementation of automated monitoring. As a result, an additional 14.5% and 17.9% fewer readings were below the BP cut points of 140/90 mm Hg and 130/80 mm Hg, respectively, and compared with the manual measurement period, the prevalence of a diagnosis of hypertension in the patient records was 4.3 percentage points higher during the automated measurement period (23.4% vs 19.1%).

HealthPartners was not the only medical group to experience an increase in mean BP values and a decrease in apparent hypertension control rates after the adoption of automated measurement. For example, a 2022 study from a single Veterans Affairs medical center primary care clinic found an even greater increase in mean SBP of 11.1 mm Hg and an increase in uncontrolled hypertension from 17.8% to 41.8% when automated measurement replaced manual measurement.^9^ Our findings are also consistent with those of a cluster randomized trial that found SBP to be 7.5 mm Hg lower in clinics using manual measurements than in clinics with BP measured by validated oscillometric automated monitors.^25^ Substantial differences in terminal digit preference between the manual and automated periods were also present for SBP (71% vs 18%; *P* < .001).^25^

Our findings are consistent with the association of persistent terminal digit preference with manual BP measurement reported in both clinical trials and clinical care.^3,4,5,6,7,8,9,10,11,12^ In response, several different devices have been created to improve observer reliability in BP measurement.^2,5,26,27^ The early mechanical devices were difficult to use and required extensive training. By contrast, more recent automated monitors such as the Omron HEM-907XL are easier to use and maintain, require less frequent recalibration, and produce valid readings.^28,29,30,31,32^ Clinical trials have used the Omron HEM-907XL^33,34^ or other automated monitors^35,36^ to avoid the recording bias associated with terminal digit preference.

Failure to accurately measure BP becomes important when it results in a failure to diagnose and treat hypertension. A mean reduction in SBP of as little as 3.6 mm Hg has been shown to be associated with reduced risk of stroke and deaths from cardiovascular disease.^37^ The benefit of treatment has been shown to accrue at SBP levels of less than 120 mm Hg and DBP levels of less than 70 mm Hg.^38^

Our observation that automated readings were, on average, higher than readings obtained manually contradicts findings of a meta-analysis that measuring with an automated office BP protocol was associated with readings that were a mean of 14.5 mm Hg lower than manually measured office SBP readings.^39^ In contrast to the measurements at HealthPartners, there was no specified protocol in that study for the routine office measurements and the type of device (manual vs automated) was not specified or recorded. The automated office BP protocol (3-5 unattended measurements regardless of whether the first measurement was elevated) in that study also was dissimilar from the automated protocol for BP measurement at HealthPartners. Another difference may have been owed to a greater emphasis in HealthPartners clinics to achieve BP goals. Depending on the situation and the observer, BP measurement and recording technique may create an upward bias, a downward bias, or both simultaneously. Roerecke et al^39^ and the American Heart Association^40^ have suggested that averaging multiple automated office BP measurements should be the preferred method for recording BP in routine clinical practice because it reduces human error and the biases associated with the auscultatory approach.

There may be reluctance to consider that BP values obtained with manual measurement may be biased downward. Although the validity and reliability of BP measurements obtained with the Omron HEM-907XL are well documented,^29,30,31,41,42,43,44,45^ many licensed practitioners (physicians, physician assistants, and advanced practice registered nurses) believe that manual measurement of BP is highly accurate and that automated measurement is not accurate or not very accurate.^46^

Although accountability programs may be associated with improvement in care processes and outcomes,^47,48,49^ they may also be associated with unexpected consequences. For example, requiring the administration of antibiotics within 4 hours for community-acquired pneumonia has been associated with an increase in misdiagnoses.^50^ Early public reporting of cardiac surgery outcomes has been associated with surgeons declining to take on high-risk cases.^51^ Hypertension control programs have been associated with unexpected consequences.^52^ Pay-for-performance programs and other quality recognition programs may create an incentive to record a BP that meets a particular goal and thus might create a disincentive for health care organizations to adopt automated measurement of BP. These pressures may make it necessary for quality improvement programs to require measurement of BP with an automated monitor as a condition of participation.

### Limitations

This study has limitations. The BPs were collected in busy clinical settings by health care professionals who likely had varying levels of skill, attention, and commitment to the accurate measurement of BP. The method by which BP was measured (manual vs automated) was not recorded in the medical record; thus, we used the date when automated monitors were deployed in the clinics. Despite the intent of HealthPartners to adopt automated measurement as the standard in April 2012, the changes in the BP values shown in Figure 2 and eFigure 1 in the Supplement suggest that manual measurement may have persisted to some degree after April 2012. Although the data were collected from 11 different primary care clinics with separate facilities and staff, all clinics were in 1 health care organization. Also, the Omron HEM-907XL has not been validated in some subpopulations, such as patients with atrial fibrillation. Because the data we analyzed are observational, we cannot confirm which measurement method produced more accurate measures, and we cannot address whether the emphasis on accurate assessment of BP and a performance recognition program contributed to the lower BP values recorded during the manual measurement period.

## Conclusions

When aneroid sphygmomanometers were replaced by automated monitors in this quality improvement study, terminal digit preference in BP measurement decreased but was not eliminated. On average, recorded BP values increased. Although the tendency for manual BP measurement to be inaccurate has been documented for decades^1,2,3,4,5,6,7,8,9,10,11^ and automated measurement has been shown to be accurate,^29,30,31,32,41,44,45^ we could not determine which method of measurement—manual or automated—was more accurate in this study because we lacked an independent standard of measurement. If the pattern of increased BP values with automated measurement is found to be widely prevalent, there may be implications for pay-for-performance programs.

## Notes

Supplement. **eTable 1.** Mean SBP Values During the Manual Measurement and Automated Measurement Periods in 28 HealthPartners Clinics **eTable 2.** Proportion of Patients With a Diagnosis of Hypertension by Age Group and Measurement Period **eFigure 1.** Percentage of SBP Measurements With 0 as the Terminal Digit During the Manual Measurement Period (Study Months –48 to 0) and Automated Measurement Period (Study Months 0 to 36) **eFigure 2.** Distribution of SBP values at the Last Measurement of the Day During the Manual Measurement and Automated Measurement Periods for Patients With Hypertension

## References

[1] Lowe CR, McKeown T. Some sources of irregularity in the distribution of arterial pressure. In: Pemberton J, ed. Epidemiology Reports on Research and Teaching 1962. Oxford University Press; 1963:131-141.

[2] Rose G. A study of blood pressure among Negro school-children. J Chronic Dis. 1962;15:373-380. doi:10.1016/0021-9681(62)90083-8 14493819

[3] Greiver M, Kalia S, Voruganti T, . Trends in end digit preference for blood pressure and associations with cardiovascular outcomes in Canadian and UK primary care: a retrospective observational study. BMJ Open. 2019;9(1):e024970. doi:10.1136/bmjopen-2018-024970 30679298 PMC6347875

[4] Nietert PJ, Wessell AM, Feifer C, Ornstein SM. Effect of terminal digit preference on blood pressure measurement and treatment in primary care. Am J Hypertens. 2006;19(2):147-152. doi:10.1016/j.amjhyper.2005.08.016 16448884

[5] Rose GA, Holland WW, Crowley EA. A sphygmomanometer for epidemiologists. Lancet. 1964;1(7328):296-300. doi:10.1016/S0140-6736(64)92408-0 14089056

[6] Wen SW, Kramer MS, Hoey J, Hanley JA, Usher RH. Terminal digit preference, random error, and bias in routine clinical measurement of blood pressure. J Clin Epidemiol. 1993;46(10):1187-1193. doi:10.1016/0895-4356(93)90118-K 8410103

[7] Harrison WN, Lancashire RJ, Marshall TP. Variation in recorded blood pressure terminal digit bias in general practice. J Hum Hypertens. 2008;22(3):163-167. doi:10.1038/sj.jhh.1002312 18046433

[8] Wingfield D, Cooke J, Thijs L, ; Syst-Eur Investigators. Terminal digit preference and single-number preference in the Syst-Eur trial: influence of quality control. Blood Press Monit. 2002;7(3):169-177. doi:10.1097/00126097-200206000-00005 12131074

[9] Gozdecki L, Kramer H, Thomas M, . Protocol to improve hypertension management in a VA outpatient clinic. J Hum Hypertens. Published online January 24, 2022. doi:10.1038/s41371-021-00650-0 35067681

[10] Foti KE, Appel LJ, Matsushita K, Coresh J, Alexander GC, Selvin E. Digit preference in office blood pressure measurements, United States 2015-2019. Am J Hypertens. 2021;34(5):521-530. doi:10.1093/ajh/hpaa196 33246327 PMC8628654

[11] Bruce NG, Shaper AG, Walker M, Wannamethee G. Observer bias in blood pressure studies. J Hypertens. 1988;6(5):375-380. doi:10.1097/00004872-198805000-00006 3385202

[12] Thavarajah S, White WB, Mansoor GA. Terminal digit bias in a specialty hypertension faculty practice. J Hum Hypertens. 2003;17(12):819-822. doi:10.1038/sj.jhh.1001625 14704725

[13] MN Community Measurement. *2012 Health Care Quality Report*. Minnesota Legislative Reference Library. December 2012. Accessed August 1, 2022. https://www.lrl.mn.gov/docs/2015/mandated/150461.pdf22417314

[14] MN Community Measurement. *2014 Health Care Quality Report*. Minnesota Legislative Reference Library. December 2014. Accessed September 15, 2021. https://www.leg.state.mn.us/docs/2015/mandated/150463.pdf

[15] Ogrinc G, Davies L, Goodman D, Batalden P, Davidoff F, Stevens D. SQUIRE 2.0 (Standards for Quality Improvement Reporting Excellence): revised publication guidelines from a detailed consensus process. BMJ Qual Saf. 2016;25(12):986-992. doi:10.1136/bmjqs-2015-004411 26369893 PMC5256233

[16] Marti-Soler H, Gubelmann C, Aeschbacher S, . Seasonality of cardiovascular risk factors: an analysis including over 230 000 participants in 15 countries. Heart. 2014;100(19):1517-1523. doi:10.1136/heartjnl-2014-305623 24879630

[17] Gepts T, Nguyen AM, Cleland C, Wu W, Pham-Singer H, Shelley D. Accounting for blood pressure seasonality alters evaluation of practice-level blood pressure control intervention. Am J Hypertens. 2020;33(3):220-222. doi:10.1093/ajh/hpz179 31711219

[18] Narita K, Hoshide S, Kario K. Seasonal variation in blood pressure: current evidence and recommendations for hypertension management. Hypertens Res. 2021;44(11):1363-1372. doi:10.1038/s41440-021-00732-z 34489592

[19] Kollias A, Kyriakoulis KG, Stambolliu E, Ntineri A, Anagnostopoulos I, Stergiou GS. Seasonal blood pressure variation assessed by different measurement methods: systematic review and meta-analysis. J Hypertens. 2020;38(5):791-798. doi:10.1097/HJH.0000000000002355 32102047

[20] Stergiou GS, Palatini P, Modesti PA, . Seasonal variation in blood pressure: evidence, consensus and recommendations for clinical practice: consensus statement by the European Society of Hypertension Working Group on Blood Pressure Monitoring and Cardiovascular Variability. J Hypertens. 2020;38(7):1235-1243. doi:10.1097/HJH.0000000000002341 31990898

[21] Bernal JL, Cummins S, Gasparrini A. Interrupted time series regression for the evaluation of public health interventions: a tutorial. Int J Epidemiol. 2017;46(1):348-355. doi:10.1093/ije/dyw098 27283160 PMC5407170

[22] Bhaskaran K, Gasparrini A, Hajat S, Smeeth L, Armstrong B. Time series regression studies in environmental epidemiology. Int J Epidemiol. 2013;42(4):1187-1195. doi:10.1093/ije/dyt092 23760528 PMC3780998

[23] Kontopantelis E, Doran T, Springate DA, Buchan I, Reeves D. Regression based quasi-experimental approach when randomisation is not an option: interrupted time series analysis. BMJ. 2015;350:h2750. doi:10.1136/bmj.h2750 26058820 PMC4460815

[24] Warton EM. Time after time: difference-in-differences and interrupted time series models in SAS. SAS Global Forum 2020: Paper 4674-2020. 2020. Accessed July 27, 2022. https://www.sas.com/content/dam/SAS/support/en/sas-global-forum-proceedings/2020/4674-2020.pdf

[25] Nelson MR, Quinn S, Bowers-Ingram L, Nelson JM, Winzenberg TM. Cluster-randomized controlled trial of oscillometric vs manual sphygmomanometer for blood pressure management in primary care (CRAB). Am J Hypertens. 2009;22(6):598-603. doi:10.1038/ajh.2009.55 19300424

[26] Holland WW. The reduction of observer variability in the measurement of blood pressure. In: Pemberton J, ed. Epidemiology Reports on Research and Teaching 1962. Oxford University Press; 1963:271-281.

[27] Evans JG, Prior IA. Experience with the random-zero sphygmomanometer. Br J Prev Soc Med. 1970;24(1):10-15. doi:10.1136/jech.24.1.10 5435081 PMC1059218

[28] Elliott WJ, Young PE, DeVivo L, Feldstein J, Black HR. A comparison of two sphygmomanometers that may replace the traditional mercury column in the healthcare workplace. Blood Press Monit. 2007;12(1):23-28. doi:10.1097/MBP.0b013e3280858dcf 17303984

[29] Gurpreet K, Tee GH, Karuthan C. Evaluation of the accuracy of the Omron HEM-907 blood pressure device. Med J Malaysia. 2008;63(3):239-243. 19248698

[30] White WB, Anwar YA. Evaluation of the overall efficacy of the Omron office digital blood pressure HEM-907 monitor in adults. Blood Press Monit. 2001;6(2):107-110. doi:10.1097/00126097-200104000-00007 11433132

[31] Ostchega Y, Nwankwo T, Sorlie PD, Wolz M, Zipf G. Assessing the validity of the Omron HEM-907XL oscillometric blood pressure measurement device in a National Survey environment. J Clin Hypertens (Greenwich). 2010;12(1):22-28. doi:10.1111/j.1751-7176.2009.00199.x 20047626 PMC8673034

[32] Belghazi J, El Feghali RN, Moussalem T, Rejdych M, Asmar RG. Validation of four automatic devices for self-measurement of blood pressure according to the International Protocol of the European Society of Hypertension. Vasc Health Risk Manag. 2007;3(4):389-400. 17969368 PMC2291343

[33] Cushman WC, Evans GW, Byington RP, ; ACCORD Study Group. Effects of intensive blood-pressure control in type 2 diabetes mellitus. N Engl J Med. 2010;362(17):1575-1585. doi:10.1056/NEJMoa1001286 20228401 PMC4123215

[34] Wright JT Jr, Williamson JD, Whelton PK, ; SPRINT Research Group. A randomized trial of intensive versus standard blood-pressure control. N Engl J Med. 2015;373(22):2103-2116. doi:10.1056/NEJMoa1511939 26551272 PMC4689591

[35] Hansson L, Zanchetti A, Carruthers SG, ; HOT Study Group. Effects of intensive blood-pressure lowering and low-dose aspirin in patients with hypertension: principal results of the Hypertension Optimal Treatment (HOT) randomised trial. Lancet. 1998;351(9118):1755-1762. doi:10.1016/S0140-6736(98)04311-6 9635947

[36] Benavente OR, Coffey CS, Conwit R, ; SPS3 Study Group. Blood-pressure targets in patients with recent lacunar stroke: the SPS3 randomised trial. Lancet. 2013;382(9891):507-515. doi:10.1016/S0140-6736(13)60852-1 23726159 PMC3979302

[37] Sundström J, Arima H, Jackson R, ; Blood Pressure Lowering Treatment Trialists’ Collaboration. Effects of blood pressure reduction in mild hypertension: a systematic review and meta-analysis. Ann Intern Med. 2015;162(3):184-191. doi:10.7326/M14-0773 25531552

[38] Blood Pressure Lowering Treatment Trialists’ Collaboration. Age-stratified and blood-pressure-stratified effects of blood-pressure-lowering pharmacotherapy for the prevention of cardiovascular disease and death: an individual participant-level data meta-analysis. Lancet. 2021;398(10305):1053-1064. doi:10.1016/S0140-6736(21)01921-8 34461040 PMC8473559

[39] Roerecke M, Kaczorowski J, Myers MG. Comparing automated office blood pressure readings with other methods of blood pressure measurement for identifying patients with possible hypertension: a systematic review and meta-analysis. JAMA Intern Med. 2019;179(3):351-362. doi:10.1001/jamainternmed.2018.6551 30715088 PMC6439707

[40] Muntner P, Shimbo D, Carey RM, . Measurement of blood pressure in humans: a scientific statement from the American Heart Association. Hypertension. 2019;73(5):e35-e66. doi:10.1161/HYP.0000000000000087 30827125 PMC11409525

[41] El Feghali RN, Topouchian JA, Pannier BM, El Assaad HA, Asmar RG; European Society of Hypertension. Validation of the OMRON M7 (HEM-780-E) blood pressure measuring device in a population requiring large cuff use according to the International Protocol of the European Society of Hypertension. Blood Press Monit. 2007;12(3):173-178. doi:10.1097/MBP.0b013e3280b08367 17496467

[42] D’Sa L, Senaratne N, Woodcock-Smith J, Miles KM, Wilkinson IB, McEniery CM. Evaluation of the Omron HEM-907 automated blood pressure device: comparison with office and ambulatory blood pressure readings. Hypertens Res. 2019;42(1):52-58. doi:10.1038/s41440-018-0120-7 30374040

[43] Cho K, Tian M, Lan Y, Zhao X, Yan LL. Validation of the Omron HEM-7201 upper arm blood pressure monitor, for self-measurement in a high-altitude environment, according to the European Society of Hypertension International Protocol revision 2010. J Hum Hypertens. 2013;27(8):487-491. doi:10.1038/jhh.2013.4 23466876 PMC3709008

[44] Omboni S, Riva I, Giglio A, Caldara G, Groppelli A, Parati G. Validation of the Omron M5-I, R5-I and HEM-907 automated blood pressure monitors in elderly individuals according to the International Protocol of the European Society of Hypertension. Blood Press Monit. 2007;12(4):233-242. doi:10.1097/MBP.0b013e32813fa386 17625396

[45] El Assaad MA, Topouchian JA, Darné BM, Asmar RG. Validation of the Omron HEM-907 device for blood pressure measurement. Blood Press Monit. 2002;7(4):237-241. doi:10.1097/00126097-200208000-00006 12198340

[46] Green BB, Anderson ML, Ehrlich K, . Blood pressure checks for diagnosing hypertension: health professionals’ knowledge, beliefs, and practices. J Am Board Fam Med. 2022;35(2):310-319. doi:10.3122/jabfm.2022.02.210318 35379718 PMC9621313

[47] Foti K, Auerbach J, Magnan S. Improving hypertension control population-wide in Minnesota. J Public Health Manag Pract. 2018;24(5):432-439. doi:10.1097/PHH.0000000000000590 28628583

[48] Wasfy JH, Hidrue MK, Ngo J, . Association of an acute myocardial infarction readmission-reduction program with mortality and readmission. Circ Cardiovasc Qual Outcomes. 2020;13(5):e006043. doi:10.1161/CIRCOUTCOMES.119.006043 32393130 PMC7237309

[49] Petersen LA, Simpson K, Pietz K, . Effects of individual physician-level and practice-level financial incentives on hypertension care: a randomized trial. JAMA. 2013;310(10):1042-1050. doi:10.1001/jama.2013.276303 24026599 PMC4165573

[50] Kanwar M, Brar N, Khatib R, Fakih MG. Misdiagnosis of community-acquired pneumonia and inappropriate utilization of antibiotics: side effects of the 4-h antibiotic administration rule. Chest. 2007;131(6):1865-1869. doi:10.1378/chest.07-0164 17400668

[51] Wasfy JH, Borden WB, Secemsky EA, McCabe JM, Yeh RW. Public reporting in cardiovascular medicine: accountability, unintended consequences, and promise for improvement. Circulation. 2015;131(17):1518-1527. doi:10.1161/CIRCULATIONAHA.114.014118 25918041

[52] Hysong SJ, SoRelle R, Broussard Smitham K, Petersen LA. Reports of unintended consequences of financial incentives to improve management of hypertension. PLoS One. 2017;12(9):e0184856. doi:10.1371/journal.pone.0184856 28934258 PMC5608267

