# Supplementary material for: Association of an Automated Blood Pressure Measurement Quality Improvement Program With Terminal Digit Preference and Recorded Mean Blood Pressure in 11 Clinics
Source: JAMA Netw Open. 2022 Aug 31;5(8):e2229098. doi: 10.1001/jamanetworkopen.2022.29098 (PMC9434355; doi:10.1001/jamanetworkopen.2022.29098)
Supplement: Supplement. — eTable 1. Mean SBP Values During the Manual Measurement and Automated Measurement Periods in 28 HealthPartners Clinics eTable 2. Proportion of Patients With a Diagnosis of Hypertension by Age Group and Measurement Period eFigure 1. Percentage of SBP Measurements With 0 as the Terminal Digit During the Manual Measurement Period (Study Months –48 to 0) and Automated Measurement Period (Study Months 0 to 36) eFigure 2. Distribution of SBP values at the Last Measurement of the Day During the Manual Measurement and Automated Measurement Periods for Patients With Hypertension [file jamanetwopen-e2229098-s001.pdf]

Supplementary Online Content

Kottke TE, Anderson JP, Zillhardt JD, et al. Association of an automated blood pressure measurement quality improvement program with terminal digit preference and recorded mean blood pressure in 11 clinics. *JAMA Netw Open*. 2022;5(8):e2229098. doi:10.1001/jamanetworkopen.2022.29098

eTable 1. Mean SBP Values During the Manual Measurement and Automated Measurement Periods in 28 HealthPartners Clinics
eTable 2. Proportion of Patients With a Diagnosis of Hypertension by Age Group and Measurement Period
eFigure 1. Percentage of SBP Measurements With 0 as the Terminal Digit During the Manual Measurement Period (Study Months –48 to 0) and Automated Measurement Period (Study Months 0 to 36)
eFigure 2. Distribution of SBP Values at the Last Measurement of the Day During the Manual Measurement and Automated Measurement Periods for Patients With Hypertension

This supplementary material has been provided by the authors to give readers additional information about their work.

© 2022 Kottke TE et al. *JAMA Network Open*.

eTable 1: Mean SBP Values During the Manual Measurement and Automated Measurement Periods in 28 HealthPartners Clinics[a]

**Clinic number**	**1**	**2**	**3**	**4***	**5***	**6**	**7***	**8***	**9***	**10***
Manual measurement period	127.4	122.8	123.7	121.4	121.4	119.2	122.1	120.9	118.2	127.1
Automated measurement period	133.1	127.3	128.7	126.2	127.2	124.0	127.2	123.2	128.0	127.0
P-Value	<.0001	<.0001	<.0001	<.0001	<.0001	<.0001	<.0001	<.0001	<.0001	0.901
**Clinic number**	**11***	**12***	**13***	**14**	**15**	**16**	**17**	**18**	**19**	
Manual measurement period	119.6	117.4	121.6	118.6	122.6	121.0	124.5	130.6	122.7	
Automated measurement period	125.3	125.7	124.8	126.4	128.2	125.4	126.2	131.0	131.1	
P-Value	<.0001	<.0001	<.0001	<.0001	<.0001	<.0001	<.0001	0.010	<.0001	
**Clinic number**	**20**	**21***	**22***	**23**	**24**	**25**	**26**	**27**	**28**	
Manual measurement period	118.3	122.4	120.6	121.6	121.8	125.4	128.7	124.4	130.2	
Automated measurement period	121.8	127.9	127.1	126.6	127.9	130.3	129.4	131.5	132.8	
P-Value	<.0001	<.0001	<.0001	<.0001	<.0001	<.0001	0.049	<.0001	<.0001	

[a]Asterisks indicate the clinics that are included in the detailed analysis

© 2022 Kottke TE et al. *JAMA Network Open*.

eTable 2: Proportion of Patients With a Diagnosis of Hypertension by Age Group and Measurement Period

	No. (%)	
Number and percent of patients with diagnosis of hypertension. Total, and by age group	MANUAL PERIOD (2008-2012)	AUTOMATED PERIOD (2012-2015)
Total	31,591 (19.1)	34,826 (23.4)
18-39	2,933 (3.9)	3,364 (5.1)
40-49	5,263 (16.7)	5,311 (20.6)
50-59	9,645 (30.4)	10,197 (35.4)
60-69	9,206 (46.7)	10,924 (51.0)
70-75	4,544 (62.0)	5,030 (67.0)

© 2022 Kottke TE et al. *JAMA Network Open*.

eFigure 1: Percentage of SBP Measurements With 0 as the Terminal Digit During the Manual Measurement Period (Study Months –48 to 0) and Automated Measurement Period (Study Months 0 to 36)

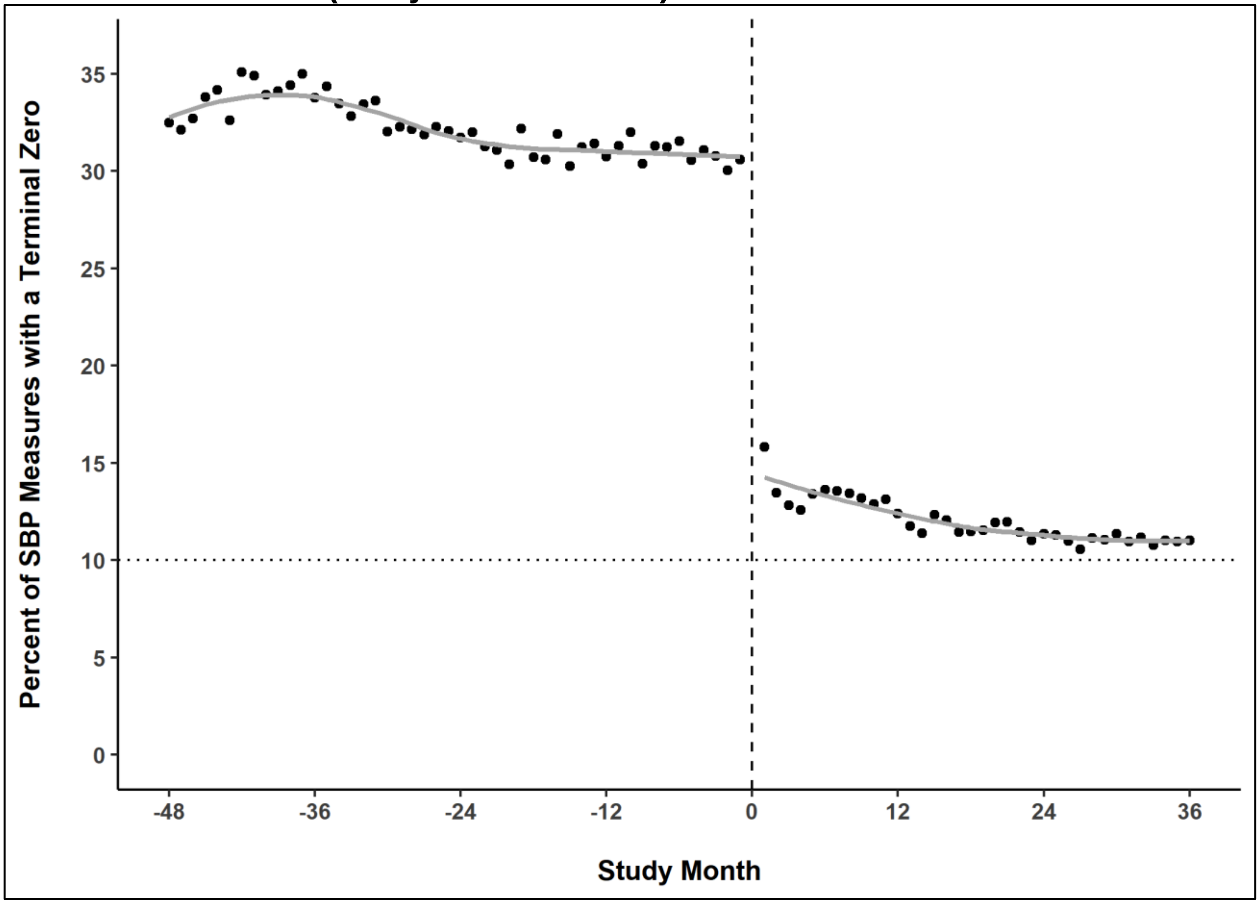

© 2022 Kottke TE et al. *JAMA Network Open*.

eFigure 2: Distribution of SBP Values at the Last Measurement of the Day During the Manual Measurement and Automated Measurement Periods for Patients With Hypertension

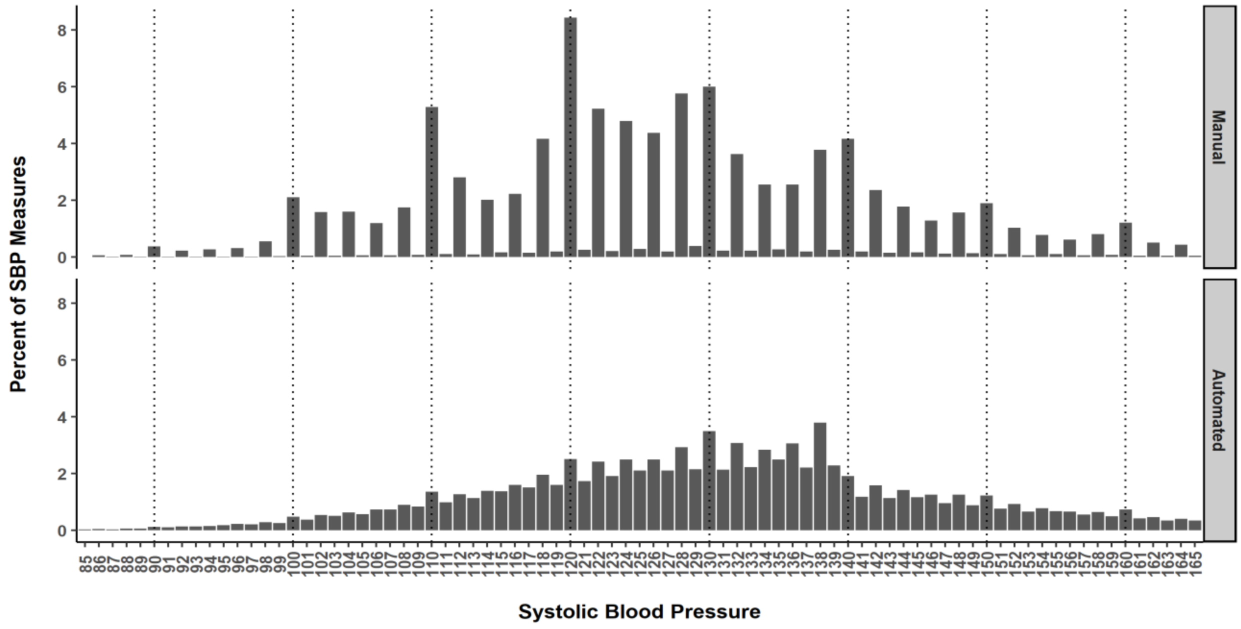

© 2022 Kottke TE et al. *JAMA Network Open*.
